# Rapid Screening of Etomidate and Its Analogs in Seized e-Liquids Using Thermal Desorption Electrospray Ionization Coupled with Triple Quadrupole Mass Spectrometry

**DOI:** 10.3390/toxics12120884

**Published:** 2024-12-05

**Authors:** Meng Li, Bicheng Lin, Binling Zhu

**Affiliations:** 1 Department of Forensic Science, Fujian Police College, Fuzhou 350007, China; limeng@fjpsc.edu.cn; 2 Fujian Zhengzhong Forensic Sciences Institute, Fuzhou 350108, China; lbc78096727@163.com

**Keywords:** e-liquid, etomidate, metomidate, isopropoxate, TD-ESI/MS/MS, screening

## Abstract

The growing popularity of e-cigarettes has raised significant concerns about the safety and potential abuse of these products. Compounds originally used in the medical field, such as etomidate, metomidate, and isopropoxate, have been illegally added to e-liquids, posing substantial risks to consumer health, and facilitating the misuse of illicit drugs. To address these concerns, this study developed a rapid and efficient method for detecting etomidate, metomidate, and isopropoxate in e-liquids using thermal desorption electrospray ionization coupling triple quadrupole mass spectrometry (TD-ESI/MS/MS). The TD-ESI/MS/MS method exhibits high sensitivity, with detection limits for etomidate, metomidate, and isopropoxate reaching 3 ng/mL. Screening of 70 seized e-liquid samples from 12 cases using TD-ESI/MS/MS revealed that 46 samples contained only etomidate, 13 samples contained only isopropoxate, and 11 samples contained both etomidate and metomidate. The qualitative results obtained from TD-ESI/MS/MS were in complete agreement with those of GC-MS. Moreover, the TD-ESI/MS/MS method requires no pre-treatment steps and has a detection time of only 1 min, thereby saving experimental consumables and significantly reducing detection time. The method demonstrated high sensitivity, accuracy, and reproducibility, making it suitable for high-throughput screening in forensic and regulatory settings.

## 1. Introduction

With the growing popularity of e-cigarette products worldwide, their safety issues have increasingly become a focal point for both the public and regulatory agencies [1,2]. Some compounds originally used in the medical field, such as etomidate, metomidate, and isopropoxate, had been illegally added to e-liquids, which could not only pose significant risks to consumer health but also facilitate the abuse of illicit drugs [3,4]. Etomidate (R-(+)-ethyl-1-(1-phenylethyl)-1H-imidazole-5-carboxylate) had been recognized as a non-barbiturate anesthetic, characterized by a rapid onset of hypnosis and minimal adverse effects [5]. In addition to this, due to its similar efficacy and usage compared to propofol, etomidate was referred to as “an alternative to propofol” [6]. Metomidate (methyl 1-(1-phenylethyl)-1*H*-imidazole-5-carboxylate) and isopropoxate (isopropyl 1-(1-phenylethyl)-1*H*-imidazole-5-carboxylate), as analogs of etomidate, exhibited similar pharmacological effects, including induction of anesthesia and sedation, comparable to those of etomidate [7]. Long-term intake of large amounts of these substances might lead to a range of mental health issues, including paranoia, anxiety, panic, and victimization delusions [8]. Such conditions could easily result in self-harm, injuries, or traffic accidents, posing significant risks to both individual physical and mental health and to public safety [6,9]. It was reported that a paramedic became ‘temporarily incapacitated’ after injecting herself twice with etomidate and using other drugs like morphine, Demerol, and Ativan [10]. A 22-year-old female of Korean descent was discovered unconscious in a bathtub following the ingestion of etomidate [11]. Recently, the illegal sale and distribution of etomidate injections have emerged as social issues in China. The China National Medical Products Administration has approved the control of several new illicit drugs, including etomidate, metomidate and isopropoxate, in an effort to intensify anti-drug operations and halt the spread of new narcotics abuse. Therefore, it is crucial and of significant forensic value to establish a reliable analytical method for the determination of etomidate and its analogs in e-liquids.

Methods for the detection of the illicit drugs in e-liquid samples by gas chromatography–mass spectrometry (GC-MS) [12,13,14], and ultraperformance liquid chromatography–mass spectrometry (UPLC−MS) [15,16] have been studied and published. However, the procedures for sample preparation continue to be characterized by significant manual effort and prolonged durations. Additionally, the extended analytical times required for GC-MS and LC-MS are less favorable for large-scale screening of seized sample sets [17].

Thermal desorption electrospray ionization coupling with triple quadrupole mass spectrometry (TD-ESI/MS/MS) represents an ambient ionization method capable of swift chemical analysis, eliminating the need for sample pre-treatment or chromatographic processes [18]. TD-ESI/MS/MS method employed a probe for the extraction of small amounts of compounds from solid or liquid samples. The metal probe was subsequently inserted into a thermal desorption device, where the analyte was thermally desorbed. The desorbed analytes were then carried through a stream of nitrogen into the electrospray ionization plume [19,20]. TD-ESI/MS/MS has been utilized to rapid screening of trace analytes, including explosives [21,22], pesticides [23,24], lipids [25,26], drugs [27,28,29], and illicit drugs (amphetamine, cocaine, ketamine, and methamphetamine) [30,31,32]. In addition to this, illicit drugs in e-liquids have been rapidly analyzed via probe sampling followed by TD-ESI/MS/MS to quickly determine whether a suspect was involved in drug abuse, which was especially important for on-site law enforcement.

To the best of our knowledge, a simultaneous analytical method for the rapid determination of etomidate and its analogs in e-liquid samples has not been established or reported. Therefore, this study was designed to establish an analytical method for the simultaneous screening of etomidate, metomidate, and isopropoxate in e-liquid samples by TD-ESI/MS/MS. In order to verify the effectiveness of TD-ESI/MS/MS method, it was applied to 70 seized e-liquid samples from 12 different cases. The results were then compared with those obtained by GC-MS method for qualitative analysis.

## 2. Materials and Methods

### 2.1. Chemicals and Reagents

Etomidate (≥98%, w%), metomidate (≥98%, w%) and isopropoxate (≥98%, w%) were supplied by Yuansi Standard Science and Technology Co. (Shanghai, China). Methanol and acetonitrile, both of LC-MS quality, were supplied by Fisher Chemical (Waltham, MA, USA). The deionized water used was obtained from a Milli-Q water purification system provided by Millipore (Billerica, MA, USA). Blank e-liquid samples were provided by the Fujian Provincial Tobacco Monopoly Bureau (Fuzhou, China). Case e-liquid samples were obtained from Fujian Fuzhou Public Security Bureau (Fuzhou, China).

### 2.2. Standard Solution

The standard stock solutions (1 mg/mL) of etomidate, metomidate and isopropoxate were prepared by dissolving in methanol and stored at 4 °C. Etomidate, metomidate and isopropoxate were diluted in methanol to achieve working solutions concentration range of 10, 20, 100, 500 ng/mL and 2, 20, 50, 100, 200, 500 µg/mL.

### 2.3. Preparation for Samples

#### 2.3.1. Preparation for Blank Samples

Blank e-liquid samples from 10 different manufacturers were mixed and diluted 1:1000 or 1:1,000,000 with methanol, respectively, to serve as the GC-MS method blank e- liquid (Blank 1) or TD-ESI/MS/MS method blank e- liquid (Blank 2). Methanol was chosen as the solvent for preparing solutions because it helped to minimize matrix effects by effectively dissolving the analytes and reducing interference from other components in the e-liquid samples.

#### 2.3.2. Preparation for Spiked e-Liquid Samples

**Figure 1:**
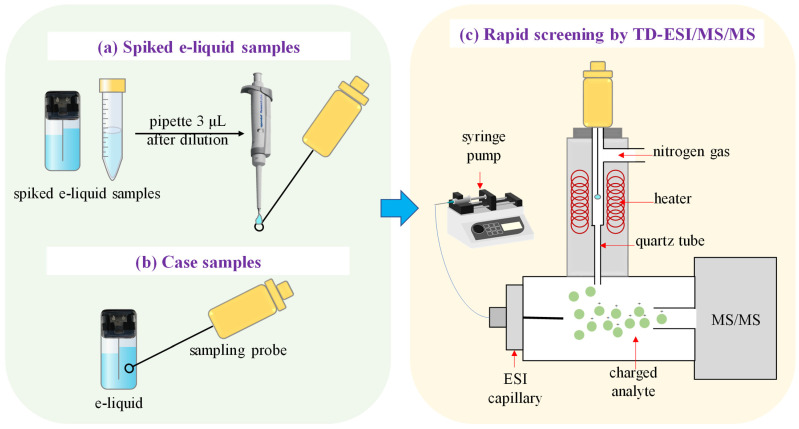
Schematic diagram of the rapid screening of etomidate and its analogs in e-liquid by TD-ESI/MS/MS: (**a**,**b**) sampling, (**c**) TD-ESI-MS analysis.

Standard stock solutions of etomidate, metomidate, and isopropoxate were added in Blank 2, to prepare spiked e-liquid samples with concentrations of 10, 20, 100 and 500 ng/mL. In total, 3 μL of spiked e-liquid sample was placed on a metal probe sampling ring and the solvent was evaporated for use in the TD-ESI/MS/MS method (Figure 1a). The appropriate volume of standard stock solutions was spiked in Blank 1 to make calibrators at 2, 20, 50, 100, 200 and 500 µg/mL for etomidate, metomidate, and isopropoxate. The analyses of spiked e-liquid samples with etomidate, metomidate, and isopropoxate were performed using a GC-MS instrument.

#### 2.3.3. Preparation for Case Samples

The metal probe sampler was used to scrape the seized electronic cigarette close to the outer wall of the sealing place 3 cm, and the sample was directly injected for rapid screening by TD-ESI/MS/MS method (Figure 1b). 10 μL of seized e-liquid was diluted with methanol to 1 mL for qualitative and quantitative analysis by GC-MS.

### 2.4. Instrumentation Condition

#### 2.4.1. TD-ESI/MS/MS

Analysis was performed on a thermal desorption electrospray ionization (Hongji Testing Technology Co., Shanghai, China) coupled with triple quadrupole mass spectrometry (Agilent, Santa Clara, CA, USA). The schematic diagram of the rapid screening of etomidate and its analogs in e-liquid by TD-ESI/MS/MS was shown in Figure 1. It was operated in multiple reaction monitoring (MRM) mode. The ion source conditions were as follows: syringe pump flow rate, 200 μL/h; heat desorption temperature, 260 °C; syringe pump solvent, 0.1% formic acid in water (containing 10 mmol/L ammonium formate)-acetonitrile (1:1, *v*/*v*). The voltage of the MS capillary was set at 4 kV, and the drying gas temperature was 300 °C with a constant flow rate of 3 L/min. The metallic sampling probe consists of a nickel–chromium alloy, featuring a wire thickness of 0.6 mm, a loop diameter of 2 mm, an overall length ranging from 5 to 6 cm, and a protruding section that extends 4 cm beyond the sampler.

#### 2.4.2. GC-MS

The e-liquid samples were performed using an Agilent 8890 gas chromatograph with 5977 MS detector (Agilent, Santa Clara, CA, USA). The separation of the extracted compounds was carried out using a DB-5MS capillary column (30.0 m × 0.25 mm × 0.25 μm). The column was initially maintained at 100 °C for 2 min. The temperature was then increased to 280 °C for 6 min at a rate of 20 °C/min. A carrier gas consisting of helium (purity 99.99%) was utilized at a steady flow rate of 1.0 mL/min, while a sample volume of 1 µL was introduced into the system operating in coupled configuration at a temperature of 250 °C. For the electron ionization process, an energy level of 70 eV was applied, with the ion source maintained at 230 °C, and the quadrupole mass filter was operated at 150 °C. The initial solvent delay was established for 3 min. The mass spectrometry data were acquired over the *m*/*z* ratio from 40 to 500 amu using full-scan detection, and the identification of substances was validated through comparison with authenticated reference standards.

### 2.5. Method Validation

The described TD-ESI/MS/MS method was validated through the analysis of selectivity, limit of detection (LOD), intra- and inter-day precision and accuracy as well as matrix effects in accordance with previous studies [33,34]. The blank e-liquid samples were subjected to TD-ESI/MS/MS analysis to investigate potential interferences that could arise during the MRM transitions for etomidate, metomidate, and isopropoxate. Three points of etomidate, metomidate, and isopropoxate in e-liquid samples at low, medium, and high concentrations (20, 100 and 500 ng/mL) between calibration ranges were analyzed to evaluate accuracy (% bias), precision (% CV) and matrix interferences of this method. The intra-day precision was evaluated by performing six replicate analyses on a single day (*n* = 6), while the inter-day precision was assessed through daily replicate analyses over a period of six consecutive days (*n* = 6). The limit of detection (LOD) was determined by spiking samples with low concentrations of the target analytes (10 ng/mL) and calculating the signal-to-noise ratio, with an LOD established at a ratio of S/N = 3.

The described GC/MS method was validated through the analysis of linearity, LOD, lower limit of quantification (LLOQ), precision, and accuracy. Qualitative results were confirmed by comparing with the retention time of chromatogram and mass spectrometry of the standard substance. Spiked e-liquid samples with concentrations of 20, 100, 500 μg/mL for etomidate, metomidate and isopropoxate were analyzed to evaluate accuracy (% bias), precision (% CV) of this method. The linearity of this method was assessed across five concentration levels (*n* = 6 replicates per level), comprising 20, 50, 100, 200, and 500 μg/mL of each analyte, with a correlation coefficient (r) exceeding 0.99. LOD and LLOQ were determined by spiking samples with low concentrations of the target analytes (2 μg/mL). LLOQ was established based on a signal-to-noise ratio (S/N) of 10. The LOD and LLOQ calculations were performed in accordance with the methods described in the previous study [35].

## 3. Results

### 3.1. Optimization of Mass Spectrometry of TD-ESI/MS/MS

Within the *m*/*z* range of 50 to 500, appropriate precursor ions were identified in the initial quadrupole using full scan mode, and suitable product ions were selected in single ion monitoring (SIM). The fragmentor range of 50–200 eV and collision energy range of 5–70 eV were investigated in product ion and MRM mode to select the optimal fragmentor and collision energy. Table 1 provided the MRM transitions along with their respective optimal collision energies.

### 3.2. Validation Results of Method

**Figure 2:**
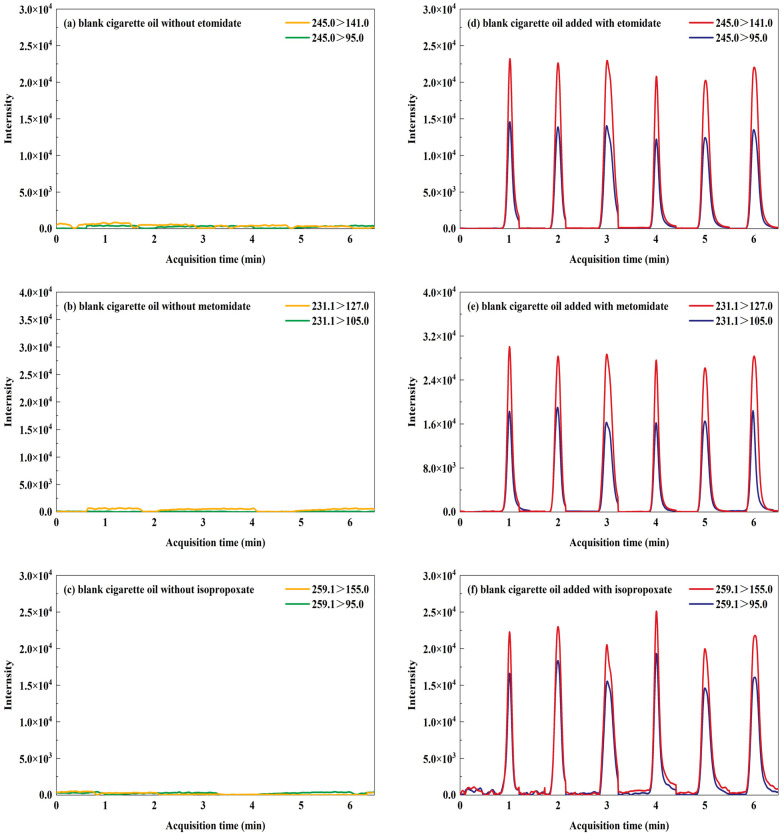
MRM chromatograms of blank e-liquid samples (**a**–**c**), blank e-liquid samples added with 100 ng/mL etomidate (**d**), metomidate (**e**), and isopropoxate (**f**).

**Figure 3:**
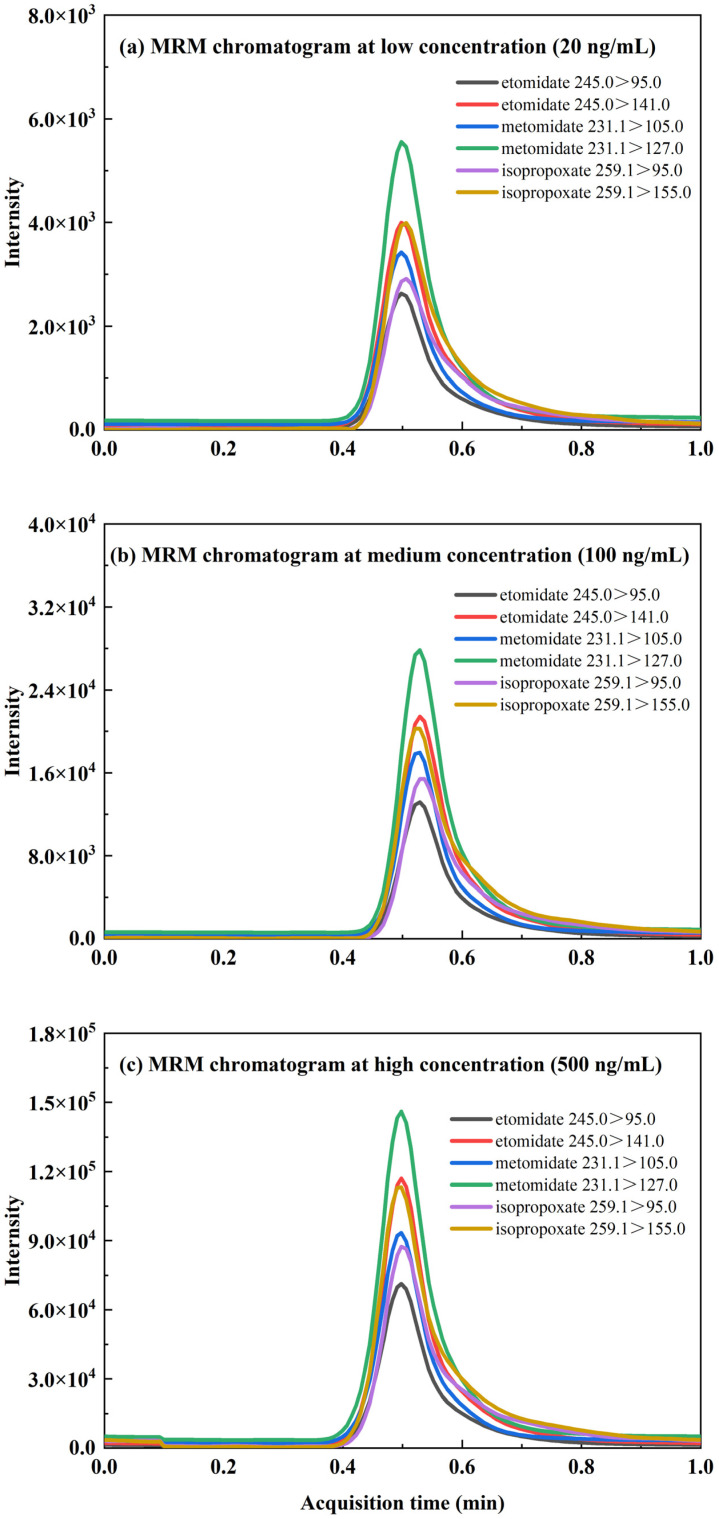
MRM chromatograms of etomidate, metomidate and isopropoxate in e-liquid samples at low (**a**), medium (**b**), and high concentrations (**c**).

Representative MRM chromatograms for the target transitions in both blank e-liquid samples and e-liquid samples spiked with etomidate, metomidate, and isopropoxate are shown in Figure 2. Notably, no interfering peaks for etomidate, metomidate, or isopropoxate were observed in the blank e-liquid samples, which underscores the high selectivity and specificity of the TD-ESI/MS/MS method for analyzing these compounds in e-liquid samples (Figure 2a–c). The MRM spectra obtained from six consecutive injections of blank e-liquid samples spiked with etomidate, metomidate and isopropoxate are presented in Figure 2d–f, with an average analysis time of 40 to 50 s, indicating that TD-ESI/MS/MS method is capable of rapidly screening for etomidate, metomidate and isopropoxate. The MRM chromatograms for etomidate, metomidate, and isopropoxate in e-liquid samples at low, medium, and high concentrations (20, 100 and 500 ng/mL) were shown in Figure 3. The peaks for etomidate, metomidate, and isopropoxate were clearly visible and distinct at all three concentrations, indicating that the TD-ESI/MS/MS method could detect these compounds with high selectivity and specificity.

As shown in Table 2, the inter-day and intra-day precision of etomidate in e-liquid samples were less than 19.1% and 16.7%, respectively; the inter-day and intra-day accuracy ranged from −4.2% to 9.5% and from −0.3% to 8.2%, respectively. For metomidate in e-liquid samples, the inter-day and intra-day precision were below 19.9% and 17.1%, respectively, with inter-day and intra-day accuracy ranging from −15.1% to 5.6% and from −16.1% to 4.0%, respectively. In the case of isopropoxate in e-liquid samples, the inter-day and intra-day precision were less than 11.5% and 12.6%, respectively, and the inter-day and intra-day accuracy were within the ranges of −8.4% to 13.1% and −11.8% to 13.6%, respectively. These results suggest that the developed method for the analysis of etomidate, metomidate and isopropoxate in e-liquid samples could demonstrate good reproducibility. The matrix effect values for etomidate ranged from 2.0% to 12.7%, for metomidate from 1.8% to 16.5%, and for isopropoxate from 1.9% to 19.5%. The LODs of etomidate, metomidate and isopropoxate detected by TD-ESI/MS/MS method were 3 ng/mL. Previous studies [36,37] have reported that the LODs and LLOQs of illicit drugs in e-liquids. The LOD and LOQ of tetrahydrocannabinol in CBD e-liquids detected by LC-HRAM-MS method were 1 μg/g and 5 μg/g, respectively [36]. Liu et al. reported that the LOQ of synthetic cannabinoid was 25 ng/mL by GC-MS method [37]. The highly sensitive instrumental analysis for TD-ESI/MS/MS method enables simplified pre-treatment procedures, facilitating rapid on-site screening of e-liquids.

To verify the reliability of the TD-ESI/MS/MS method, the rapid screening of seized e-liquid samples using TD-ESI/MS/MS was subsequently confirmed by GC-MS. The qualitative and quantitative capabilities of the GC-MS instrument were therefore examined, and the results were presented in Table 3. The R^2^ values for etomidate, metomidate, and isopropoxate were all greater than 0.997 within the concentration range of 20–500 μg/mL. The LODs and LLOQs were 0.3 μg/mL and 1 μg/mL, respectively. The intra- and inter-day accuracy and precision, assessed at low, medium, and high concentrations, were all within acceptable ranges.

### 3.3. The Result of Application Case

**Figure 4:**
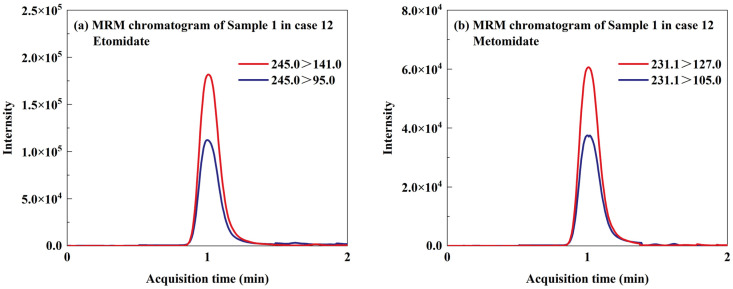
MRM chromatograms of Sample 1 in case 12.

**Figure 5:**
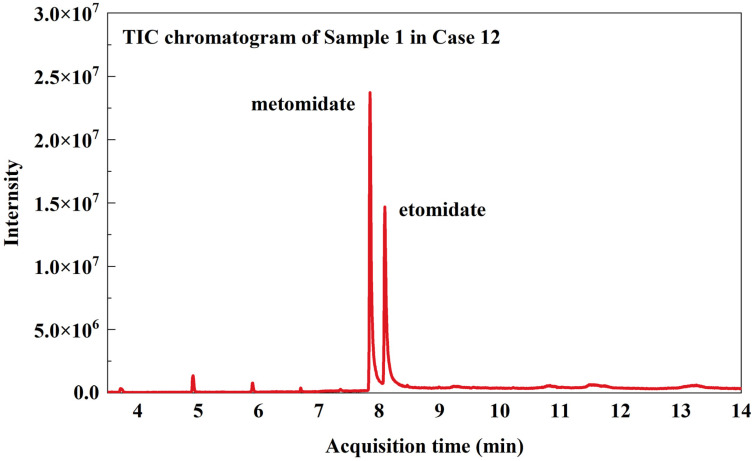
The TIC of Sample 1 in case 12.

In 2024, the Fujian Fuzhou Public Security Bureau provided our lab with numerous bottles of e-liquid for us to conduct qualitative and quantitative analyses as part of a forensic toxicology investigation. The established TD-ESI/MS/MS method was used to rapidly screen 70 seized e-liquid samples in 12 cases. In the process of making illicit e-liquid, suspects would inevitably leave traces of illicit drugs on the surface of the cartridge due to the contact between their hands or tools and the cartridge. The TD-ESI/MS/MS method exhibits high sensitivity, with detection limits for etomidate, metomidate, and isopropoxate reaching 3 ng/mL. There was no need to disassemble the pod, and the metal probe sampling ring is directly scraped on the surface of the pod shell about 3 cm in length, and the illicit drugs distributed on the surface of the pod shell could be obtained without affecting the integrity of the sample appearance. The sampled metal probe was directly inserted into the ion source inlet for detection. After the test was completed, the probe was removed, and the whole instrument screening process was completed within 1 min. The results of the case were shown in Table 4. Screening of 70 seized e-liquid samples from 12 cases using TD-ESI/MS/MS revealed that 46 samples (65.7%) contained only etomidate, 13 samples (18.6%) contained only isopropoxate, and 11 samples (15.7%) contained both etomidate and metomidate. The qualitative results obtained from TD-ESI/MS/MS were in complete agreement with those of GC-MS. The MRM chromatogram from the TD-ESI/MS/MS method and the TIC (Total Ion Current) profile from the GC-MS method for the representative sample (Sample 1 from Case 12) were shown in Figure 4 and Figure 5, respectively. Following the extraction of quantitative ions using the GC-MS method and comparison with standards, the following quantitative results were obtained: the content of etomidate, metomidate, and isopropoxate in the seized e-liquid samples ranged from 12.1% to 27.2%, 9.9% to 13.5%, and 17.3% to 27.3%, respectively. This result indicated that the content of etomidate substances in the seized e-liquid samples is very high, and the quantitative results were 9.9–27.3%, which was related to the excellent solubility of etomidate substances in e-liquid. This finding contrasts significantly with the literature reports indicating that synthetic cannabinoids are illegally added to e-liquids at concentrations of 0.1% to 2.7% [37,38,39]. In addition, despite the e-liquid samples seized in the same case having a uniform appearance, the types and concentrations of illegally added substances varied. E-liquid containing high concentrations of etomidate substances may also be diluted by drug traffickers multiple times before being sold to abusers for consumption, and the types and contents of e-liquid obtained by abusers from different illicit sources were uncertain, which would greatly increase the risk of adverse consequences of abusing such illicit products.

### 3.4. Reusability of Metal Probe Samplers

After rapid sample screening using a metal probe sampler, the ring could be burned over a 1000 degree flame to remove residue from the surface. No contaminant interference was detected in the metal probe sampler following high-temperature burning, indicating its reusability. In this study, the metal probe sampler was used more than 100 times without any observed abnormalities. The reusability of the metal probe samplers can lead to substantial cost savings.

## 4. Discussion

Previous studies have reported several methods for the detection of illicit drugs in e-liquid. The instrumental analysis methods include UPLC−MS/MS [40], UPLC [41], LC coupled to a diode-array-detector (DAD) [42], LC-coupled to a high-resolution accurate mass spectrometer (HRAM-MS) [36], and GC-MS [37,43]. The analytes included etomidate homolog (etomidate and metomidate) [40], synthetic cannabinoids (CUMYL-PEGACLONE, MDMB-4en-PINACA, 4F-MDMB-BUTINACA, ADB-BUTINACA, ADB-BUTINACA, 4F-MDMB-BUTICA, 5F-MDMB-PICA) [37,41,43], and cannabinoids (tetrahydrocannabinol and cannabidiol) [36,42]. The e-liquid samples were diluted or extracted with methanol, n-hexane/ethyl acetate, or acetonitrile/water and subsequently processed by ultrasonic, high-speed centrifugation, or microporous membrane filtration. The detailed information about determination methods and the results from the analysis of illicit drugs in e-liquid were provided in Table 5. Compared with previous studies, this study detected etomidate homolog, including etomidate, metomidate and isopropoxate. Additionally, the TD-ESI/MS/MS method requires no pre-treatment steps and has a detection time of only 1 min, thereby saving experimental consumables and significantly reducing detection time. It should be noted that the TD-ESI/MS/MS method also had its limitations. It was more suitable for qualitative analysis and cannot provide accurate quantitative data. As a result, the positive samples identified during the rapid screening should be further confirmed by methods such as GC-MS, LC-MS/MS.

Due to its small size and the fact that the ion source works at atmospheric pressure without vacuuming, the TD-ESI/MS/MS instrument could be used not only in traditional laboratory sites, but also in vehicle-mounted mobile laboratories to work in the field of large quantities of seized substances. The advantage of using TD-ESI/MS/MS at the crime scene was that it could greatly improve work efficiency, and the preliminarily judged positive e-liquid samples could be confirmed by GC-MS in the laboratory, reducing the work pressure of the laboratory. In addition, this method could also avoid the loss and contamination of the probe in contact with other physical evidence during the inspection process and ensure the reliability of physical evidence.

## 5. Conclusions

In this study, the TD-ESI/MS/MS method was used to simultaneously determine etomidate, metomidate, and isopropoxate in e-liquid samples. Our study verified the efficacy of this approach through the analysis of selectivity, LOD, precision, accuracy, and matrix effects, with outcomes meeting all the established standards for method validation. The precisions of etomidate, metomidate, and isopropoxate in e-liquid samples were less than 19.9%. The accuracy of etomidate and its analogs ranged from −16.1% to 13.6%. Matrix effects were generally less than 19.5% and the LODs detected by TD-ESI/MS/MS method were 3 ng/mL. Previous studies have developed methods for detecting illicit drugs in e-liquid using LC-DAD, UPLC−MS/MS and GC-MS-MS/MS [36,37,40,41,42,43], which required large amounts of samples and long preparation time. In contrast, the TD-ESI/MS/MS method significantly simplified the pre-analytical procedures and required only a small amount of sample for the determination of etomidate and its analogs.

In order to assess the suitability of TD-ESI/MS/MS method, the established method was applied to 70 seized e-liquid samples from 12 cases. The results showed that 46 samples contained only etomidate, 13 samples contained only isopropoxate, and 11 samples contained both etomidate and metomidate, which were consistent with the results obtained from GC-MS analysis. These findings indicated that TD-ESI/MS/MS method could be useful in identifying etomidate and its analogs in e-liquid and providing valuable forensic evidence.

## Figures and Tables

**Table 1:** Optimized results of mass spectrometry of TD-ESI/MS/MS.

Analyte	Structural Formula	Precursor Ion (*m*/*z*)	Product Ion (*m*/*z*)	Fragmentor (eV)	CE (eV)	Polarity
Etomidate	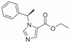	245.1	141.0	100	5	Positive
			95.0	100	25	Positive
Metomidate	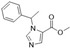	231.1	127.0	80	4	Positive
			105.0	80	28	Positive
Isopropoxate	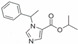	259.1	155.0	100	6	Positive
			95.0	100	28	Positive

**Table 2:** Validation results of TD-ESI/MS/MS method.

Compound	LOD (ng/mL)	Quality (ng/mL)	Inter-Day (*n*= 6)		Intra-Day (*n*= 6)		Matrix Effect (%)
			Accuracy (%)	Precision (%)	Accuracy (%)	Precision (%)	
Etomidate	3	20	9.5	19.1	8.2	16.7	12.7
		100	4.9	10.6	4.5	9.4	4.3
		500	−4.2	6.2	−0.3	7.0	2.0
Metomidate	3	20	−15.1	19.9	−16.1	17.1	16.5
		100	5.6	12.6	4.0	13.9	5.3
		500	−2.7	5.7	−0.4	5.1	1.8
Isopropoxate	3	20	13.1	11.5	13.6	8.3	19.5
		100	−8.4	7.6	−11.8	12.6	6.4
		500	−4.1	10.1	0.1	7.5	1.9

**Table 3:** Validation results of GC-MS method.

Compound	RT (min)	Precursor Ion (*m*/*z*)	Product Ion (*m*/*z*)	LOD (μg/mL)	LLOQ (μg/mL)	Linearity and Range (μg/mL)	Correlation Coefficient	Concentration (μg/mL)	Inter-Day (*n* = 6)		Intra-Day (*n* = 6)	
									Accuracy (%)	Precision (%)	Accuracy (%)	Precision (%)
Etomidate	8.090	244	105 *, 77	0.3	1	20–500	0.998	20	4.5	1.3	3.9	2.6
								100	3.6	2.2	4.7	4.1
								400	3.0	2.5	4.3	4.5
Metomidate	7.845	230	105 *, 77	0.3	1	20–500	0.998	20	3.7	2.6	2.9	3.4
								100	2.3	1.5	3.3	2.8
								400	2.4	2.3	4.6	2.7
Isopropoxate	8.179	258	105 *, 77	0.3	1	20–500	0.998	20	2.1	2.2	2.8	3.0
								100	3.8	1.7	3.7	4.7
								400	1.1	1.9	2.2	2.6

* represents quantified ions.

**Table 4:** The detection results of 70 samples in 12 seized e-liquid cases.

Case No.	Sample No.	Appearance	TD-ESI/MS/MS	GC-MS	
				Qualitative Results	Quantitative Results (%)
1	1–15	2 mL of colorless liquid, packed in cartridges	etomidate	etomidate	23.8–25.9
2	1–3	2 mL of colorless liquid, packed in cartridges	etomidate	etomidate	24.9–26.9
3	1–9	2 mL of colorless liquid, packed in cartridges	etomidate	etomidate	24.4–27.2
4	1	1 mL of tawny liquid, packed in cartridges	etomidate	etomidate	23.2
5	1	1 mL of colorless liquid, packed in cartridges	etomidate	etomidate	19.2
6	1	2 mL of tawny liquid, packed in cartridges	etomidate	etomidate	12.1
7	1	1 mL of colorless liquid, packed in cartridges	etomidate	etomidate	16.9
8	14	1 mL of colorless liquid, packed in cartridges	etomidate	etomidate	18.4–20.2
9	1	2 mL of colorless liquid, packed in cartridges	isopropoxate	isopropoxate	17.3
10	1	2 mL of colorless liquid, packed in cartridges	etomidate	etomidate	24.2
11	1	1 mL of colorless liquid, packed in cartridges	isopropoxate	isopropoxate	18.5
12	1–22	2 mL of colorless liquid, packed in cartridges	1–11: etomidate and metomidate, 12–22: isopropoxate	1–11: etomidate and metomidate, 12–22: isopropoxate	1–11: etomidate 17.1–23.4, metomidate 9.9–13.5; 12–22: isopropoxate 23.5–27.3

**Table 5:** Summary determination methods of illicit drugs in e-liquid.

Pre-Treatment	Instrument	Analytes	LOD	LLOQ	Conclusions	Reference
The seized e-liquid sample was diluted with methanol.	UPLC−MS/MS	etomidate and metomidate	0.5 pg/mg	1 pg/mg	The concentrations of etomidate and metomidate in the seized e-liquid were 95.8 μg/mg and 2.8 μg/mg, respectively.	[40]
10 mg of e-liquid was added to 10 mL of methanol. The mixture was then subjected to ultrasonic extraction for 20 min. Following high-speed centrifugation, the supernatant was collected and filtered through a microporous membrane.	GC-MS	CUMYL-PEGACLONE	1 ng/mg	2 ng/mg	The mass fractions of CUMYL-PEGACLONE in the two e-liquid samples were 0.17% and 0.21%, respectively.	[43]
Dilute 20 μL of e-liquid sample with 1 mL of methanol.	GC-MS	MDMB-4en-PINACA, 4F-MDMB-BUTINACA, ADB-BUTINACA, 4F-MDMB-BUTICA, 5F-MDMB-PICA	-	0.025 mg/mL	The concentration of synthetic cannabinoids in 25 e-liquid samples ranged from 0.05% to 2.74%.	[37]
50 mg of e-liquid were added to 10 mL of methanol, shaken and thoroughly mixed, followed by ultrasonic extraction for 30 min.	UPLC	MDMB-4en-PINACA, ADB-BUTINACA, 4F-MDMB-BUTICA, 4F-ABUTINACA, 5F-MDMB-PICA	0.2 mg/L	0.6 mg/L	This method could fully isolate and quantify the five synthetic cannabinoids in e-liquid samples within 10 min.	[41]
200 μL of e-liquid was extracted with 1000 μL of n-hexane/ethyl acetate (7:3, *v*/*v*), shaken for 5 min, centrifuged at 8 °C and 17,000g for 10 min, and a 10 μL aliquot of the supernatant was diluted with 990 μL of ethyl acetate.	FTIR-UATR, LC-DAD	Tetrahydrocannabinol (THC)	-	0.001 mg/mL	All analyzed samples exhibited a total THC content of less than 0.1059% by weight.	[42]
E-liquid samples were diluted to 1:50 with acetonitrile/water (50:50).	LC- HRAM-MS	Cannabidiol and THC	1 μg/g	5 μg/g	An optimized method was developed to screen for 17 different cannabinoids in CBD e-liquids and to accurately quantify the major cannabinoids.	[36]

## Data Availability

Data are contained within the article.
